# The Effects Of Right Ventricular Apical Pacing On Left Ventricular Function. *Stimulation Of The Right Ventricular Apex: Should It Still Be The Gold Standard?*

**Published:** 2003-04-01

**Authors:** T Szili-Torok, A Thornton

**Affiliations:** Department of Cardiology, Thoraxcentre, Erasmus MC, Rotterdam, The Netherlands

**Keywords:** left ventricular function, pacing

## Abstract

Current pacing practice is undergoing continuous and substantial changes. Initially pacing had an exclusively palliative role, since it was reserved for patients developing complete heart block or severe symptomatic bradycardia. With the appearance of novel pacing indications such as pacing for heart failure and atrial fibrillation, the effect of pacing site on cardiac function has become a critically important issue and a subject for consideration. It seems that the classical pacing site in the right ventricular apex is no longer the gold standard because of possible disadvantageous effects on cardiac function. The aim of this review article is to discuss the effect of right ventricular apical pacing on cardiac function including cellular and hemodynamic changes. We also aim to discuss the role of alternative pacing sites in the light of cardiac function.

## Background

One of the most challenging tasks of modern pacing is to optimise or at least stabilize cardiac performance. This is clearly dependent on the important factors of chronotropic function, the quality of AV synchrony, the ventricular activation sequence and ventricular pacing site. Over the last 40 years stimulation of the right ventricular apex became the standard method for pacing the ventricles because it is a stable and easily accessible site, and usually provides appropriate sensing and threshold parameters [1,2]. Although it was quickly realised that stimulation of this site leads to an abnormal contraction pattern by bypassing the physiological conduction system, for pacing in patients with life-threatening conditions it still seemed appropriate. However, with the widening of classical pacing indications and the realisation that some patients developed deterioration of left ventricular function and sometimes heart failure after pacing, it became the subject of studies into its effect on left ventricular function.

The aim of this review is to provide insights into understanding how the pacing site may influence cardiac function. This may have an impact on selection of alternative locations for the right ventricular lead when preservation or even improvement of left ventricular function seems to be important for the patient.

## Endocardial activation during right ventricular apical pacing

During ventricular pacing the impulse conduction occurs predominantly through the working myocardium and the normal conduction system is bypassed. During right ventricular pacing the right ventricle is activated first followed by trans-septal activation of the left ventricle, then the remaining part of the left ventricle is activated. The QRS duration is increased, due to a combination of slow trans-septal activation (local and diffuse), and delayed activation of the remaining part of the left ventricle [3]. A partial comparison can be drawn between the endocardial activation of patients with right ventricular apical pacing and patients with LBBB, although there are some differences. In patients with LBBB, endocardial breakthrough of right ventricular activation is on the right ventricular septum and this occurs earlier than left ventricular breakthrough [4]. This means that right ventricular activation in patients with LBBB is normal for most patients [4]. Since in most patients the right ventricular lead is placed more antero-apically than septally, clearly the endocardial activation during right ventricular apical pacing is different, resulting in a more superior axis and even more intraventricular delay during the activation of the right ventricle. On the other hand the left ventricle is still activated trans-septally in patients with right ventricular apical pacing, however left ventricular breakthrough has a heterogeneous pattern in these patients, resulting in a somewhat different activation pattern to that observed during LBBB. In most patients with LBBB high septal breakthrough is most common [4], whereas in most patients during RV apical pacing a single site of left ventricular breakthrough close to the ventricular apex is found. The presence of structural heart disease such as previous myocardial infarction may further increase the QRS duration during right ventricular apical pacing [5]. The effects of pacing site will also be different depending on whether intrinsic AV node conduction and thus fusion of activation patterns, is present [6,7].

## Effect of right ventricular apical pacing on the structure and function of the heart

For many years the attention given to optimising the atrio-ventricular delay during dual chamber pacing has delayed an understanding of the importance of the effect of pacing site on global and regional myocardial function. This is despite the fact that diminished ventricular function during pacing at the right ventricular apex has been known for decades from numerous animal and human studies [8-10]. Ventricular pacing results in an abnormal sequence of activation and this is associated with decreases in fiber shortening, contractile work, and myocardial blood flow and oxygen consumption in regions activated early and increases in these parameters in those regions with delayed activation [11-13]. Studies have also confirmed that the early-activated regions are hypofunctional and the late activated regions are hyperfunctional. These differences are due to regional alterations in effective preload during asynchronous activation. The ventricular wall has to adapt to these changes. The asynchronous activation induces asymmetrical hypertrophy of the left ventricular wall. This asymmetric hypertrophy in itself however does not primarily influence the pump function. Abnormal electrical activation may thus lead to depressed left ventricular function [12] by the classical theory of "loss of effective muscle mass". According to this theory, during ventricular pacing the ventricle looses a part of its effective muscle mass due to reduced function of the early-activated regions. An alternative or probably additional mechanism is that RV apical pacing results in inferior interventricular coupling, since during RV pacing the right ventricular pressure rises much earlier than that in the LV and this causes significant paradoxical septal motion [10].

Experimental animal data have also indicated that right ventricular apical pacing may decrease regional myocardial blood flow within the interventricular septum [14,15]. Prolonged compression of the septal arteries, as a result of the altered left ventricular depolarisation sequence was proposed as a possible explanation [14,16]. Extravascular compression is also a well-known determinant of coronary blood flow during the basal state or during coronary vasodilation [17]. There is evidence, that aberrant and delayed depolarisation of the left ventricle can result in augmented intra-myocardial pressure in the septum, thus significantly affect myocardial perfusion in this region [16]. These animal data have been confirmed by human studies, where atrial and/or AV sequential pacing did not alter coronary flow reserve, however ventricular pacing decreased resting coronary flow velocity in some patients [18]. Furthermore, long term right ventricular apical pacing results in a high incidence of myocardial perfusion defects on nuclear studies. The magnitude of these defects was linearly proportional to the duration of pacing [19]. These myocardial perfusion abnormalities are associated with apical wall motion abnormalities and impaired global left ventricular function [19]. Ventricular pacing also results in regional changes in tissue perfusion and heterogeneity between perfusion and sympathetic innervation [20]. Both ventricular and dual chamber pacing are associated with increase in cathecolamine activity [20]. Furthermore, so-called functional mitral regurgitation plays a crucial role in suboptimal hemodynamics. Right ventricular apical pacing causes significant intraventricular conduction delay. Segments of the left and right ventricle contract at different times and the interventricular septal wall contracts abnormally. This phenomenon results in decreased contractility, reduced diastolic filling and prolonged duration mitral regurgitation. The abnormal activation of the ventricles via right ventricular apical pacing may result in multiple abnormalities of cardiac function, which may ultimately affect clinical outcome.

## Chronic pacing of the right ventricular apex for symptomatic bradycardia

Acute clinical studies show that atrial pacing with preserved atrio-ventricular conduction and normal intraventricular conduction ensure better pump function at rest and a low pacing rates than DDD pacing with a restricted AV interval [21]. Because of the lack of long term follow up these findings were not confirmed by clinical studies. On the other hand evidence has been growing that pacing at alternative pacing sites improves function and influences outcome as well [22-24]. The maintenance of atrio-ventricular synchrony is a critically important issue for these patients [25]. The risk of atrial arrhythmias and thrombo-embolic complications is clearly higher in VVIR paced patients than in AV sequentially or AAI paced patients specifically if AV conduction is preserved [26]. VA conduction is has been documented in 75% of the patients with SSS and even in more than 50% of the patients with complete AV block [27]. Therefore maintenance of AV synchrony prevents "pacemaker syndrome". Improved exercise tolerance was also reported. Patients with SSS and VVI pacing are more likely to develop heart failure than with AAI pacing [26,28]. One of the key targets of pacing therapy apart from mortality is improved QOL. Initially, DDD pacing seemed to be of most benefit in patients with high degree AV block and preserved sinus node function [29]. However this hypothesis was not confirmed by recent multicenter study. Patients with SSS had more benefit from DDD pacing compared with VVI pacing, than patients with complete AV block [30]. Another important issue, that may explain the controversial clinical data is related to the duration of right ventricular apical pacing. It seems that a longer duration of right ventricular pacing results in a more myocardial perfusion abnormalities [19]. Interestingly, this phenomenon seems to be reversible even after two years pacing [19,31]. The duration of right ventricular pacing was also associated with the magnitude of decreased left ventricular ejection fraction. The reduced ejection fraction is mainly caused by an increased left ventricular end-systolic volume.

## Left ventricular function after AV node ablation and right ventricular apical pacing for patients with permanent atrial fibrillation: Discordant evolution of subjective and objective parameters

Patients with permanent atrial fibrillation undergoing AV node ablation and VVIR pacemaker implantation are optimal subjects for studying the effect of right ventricular apical pacing on left ventricular function for two reasons. First of all, these patients have no AV conduction, therefore the effect of AV delay does not influence the results; the effect of pacing site on the function could be studied independently. The other obvious reason is related to the concept of tachycardiomyopathy [32]. A relatively large proportion of these patients are suffering for a long time from permanent atrial fibrillation. Atrial fibrillation is a common supraventricular arrhythmia, which leads to cardiac dilatation and dysfunction in some patients - tachycardiomyopathy [32]. Theoretically, and practically in the majority of patients, ablation of the atrio-ventricular node followed by right ventricular apical pacing may result in an improvement of the patient's symptoms as well as in cardiac function because of the advantage of a regular ventricular response and adequate rate control [32-35]. However, variable results were reported about the course of patients following AV junction ablation. Although noticeable improvement in QOL has been reported, some other studies reported not improved or sometimes decreases left ventricular function [34,36,37]. An important aspect of these controversial data is that in most available large studies only data on the overall group was reported, despite the obvious fact that some patients deteriorated. After careful analysis of the data extracted from these studies, it seems that during the follow up, objective and subjective parameters showed somewhat of a discordant evolution. Correct interpretation of this data may allow us to develop a better understanding of the natural course of these patients and the reasons for this discordance. After AV node ablation numerous factors are influencing LV function. Some of them act in the direction of improvement, but some of them may cause deterioration. Regularisation and ventricular rate control appear to be the most important factors that may have an impact on improvement. On the other hand right ventricular apical pacing results in disadvantageous cellular changes and worsened hemodynamics. It seems so far, that the net effect of interplay between the beneficial and the worsening factors is unpredictable. The almost uniform improvement in quality of life supports the idea that subjective parameters are more influenced by the beneficial factors, however function react independently. In some patients, concordant with the QOL, function improves, however in others, despite the improvement in QOL, it may deteriorate. Therefore, in symptom control, regularisation and rate control are important factors, but their role in functional changes are not that clear. This variable outcome is of clinical significance as per the important question of Wood, as to whether AV node ablation is applicable to a wider spectrum of patients [37]. According to our experience and unpublished data we think that the effect of AV node ablation and right ventricular apical pacing on cardiac function is highly dependent on the baseline left ventricular ejection fraction. It seems that patients with preserved left ventricular function will more likely deteriorate their left ventricular function. Therefore, this therapy should be avoided in patients where only symptom control is the goal and who have normal cardiac function. This is in concordance with the concept of tachycardiomypoathy. It seems, that AV node ablation and right ventricular apical pacing is the best for patients with tachycardiomyopathy [32].

## Alternative pacing sites for preservation of left ventricular function: The possible role of high ventricular, left ventricular and biventricular pacing

As shown in previous trials, pacing of the right ventricular apex (the traditional pacing site) reduces LV function more than pacing the high ventricular septum or at LV sites [8,9,38]. Since pacing of these alternate sites results in a narrowed QRS complex as compared to RV apical pacing, synchronisation of electrical activation of the two ventricles seems to have a certain level of importance [7,24]. However, in some studies LV pacing without reduction of the QRS duration was more beneficial than biventricular pacing with marked reduction in QRS duration [10]. The study of Prinzen et al. showed improved hemodynamics with high ventricular septum pacing [6], while Blanc and colleagues demonstrated improvement with LV pacing compared with RV apical pacing [39]. Biventricular pacing had a similar effect [39]. Interestingly enough, LV pacing alone was sufficient to improve LV function [39]. We can conclude from these studies that the pacing site probably has more influence on LV function than changes in the activation sequence itself. Certainly all of these studies were acute hemodynamic studies; therefore correlation with long-term results is still to be investigated. The mechanism of this action stems from the classical theory of "loss of effective muscle mass". According to this theory, during ventricular pacing the ventricle looses a part of its effective muscle mass due to reduced function of the early activated regions. Studies confirmed that the early-activated regions are hypofunctional and the late activated muscles are hyperfunctional. This results in a substantial degree of asynchrony. An alternative mechanism is that left ventricular pacing results in a superior interventricular coupling. During RV pacing the right ventricular pressure rises much earlier than during LV pacing and causes a significant paradoxical septal motion [10].

In conclusion, the ventricular pacing site has a major effect on ventricular function. It influences hemodynamics, initiates cellular changes and alters myocardial perfusion. These changes might result in left ventricular dysfunction in a considerable number of patients. Since the patients who develop dysfunction with pacing cannot be predicted according to our present knowledge, selection of alternative pacing sites, when the preservation and improvement of left ventricular function is important, should be considered during the routine clinical practice.
